# Macroalgae-Derived Multifunctional Bioactive Substances: The Potential Applications for Food and Pharmaceuticals

**DOI:** 10.3390/foods11213455

**Published:** 2022-10-31

**Authors:** Jiameng Guo, Mei Qi, Hongyu Chen, Chengxu Zhou, Roger Ruan, Xiaojun Yan, Pengfei Cheng

**Affiliations:** 1 College of Food and Pharmaceutical Sciences, Ningbo University, Ningbo 315211, China; 2 Center for Biorefining and Department of Bioproducts and Biosystems Engineering, University of Minnesota-Twin Cities, Saint Paul, MN 55108, USA; 3 Laboratory of Marine Biological Resources and Molecular Engineering, Marine Science and Technical College, Zhejiang Ocean University, Zhoushan 316022, China

**Keywords:** macroalgae, bioactive substances, food applications, wastewater and waste gas treatment, resource utilization

## Abstract

Macroalgae, as one of the important photosynthetic organisms in the marine environment are widely used in various fields, particularly in the production of food and pharmaceuticals. Given their wide distribution, easy accessibility and high efficiency in fixing carbon dioxide through the carbon concentrating mechanism, they can produce abundant nutriments or metabolites. Moreover, macroalgae can assimilate nitrogen and phosphorus bases on the purification of wastewater, and thus further accumulate high levels of bioactive substances. This review mainly introduces the distribution characteristics of macroalgae and their unique bioactive applications in food, medicine and environmental remediation. Their functional ingredients and bioactive substances are beneficial in food production and/or medicine development. Resource utilization of macroalgae coupled with wastewater and waste gas treatment would provide a sustainable path for bioactive substances production.

## 1. Introduction

In recent years, research on sustainable bioenergy and bio-based high-value products is gaining increasing attention as the global population increases. Macroalgae are one of the most ubiquitous photosynthetic organisms worldwide and they can grow and survive under extreme conditions [1]. In general, algae are divided into microalgae and macroalgae according to their cell sizes [2]. Macroalgae have more than 1200 species and are commonly found in freshwater and marine environments. Moreover, they are essential primary producers that maintain marine ecosystems, providing more than 40% of O_2_ worldwide [3].

In detail, macroalgae are multicellular eukaryotic autotrophs on intertidal or subtidal rocky reefs with pseudo roots and viable solid growth. Their structure is complex and diverse in form and colour. Some species of macroalgae can be more than 60 m long and are the largest in shape. In general, macroalgae can be classified into three phyla based on the colour of the foliage: red algae, green algae and brown algae. Green algae produce orange-yellow pigments and contain carotene, lutein, chlorophyll a and chlorophyll b. Meanwhile, red algae (most common in hot oceans) have chlorophylls a and d and carotenoids. The staining of red algae is caused by the presence of phycoerythrin (pigment) in the cells. Brown algae contain the pigments lithophane, chlorophylls a and c, and carotenoids, it also contains oils and polysaccharides as the storage substances [4,5]. Macroalgae have the potential to accumulate bioactive compounds, which provide an extensive source for the applications of food and pharmaceuticals.

Macroalgae can be used as biological indicators of water quality, and they can greatly contribute to the global carbon, water and nutrient cycling, thereby reducing the greenhouse effect. Previous studies have shown that macroalgae treat pollutants from wastewater can sequester 10 times more carbon than other terrestrial plants [6,7,8]. Moreover, macroalgae have a high value in food production that directly affects the yield and quality of some aquatic economic animals and an important link in the biological food chain of water bodies. Some macroalgae such as *Ulva* spp. and *Artemia japonica* are rich in trace elements such as N, I, and K that can be used to feed animals and are widely processed into aquatic bio-bait. Notably, macroalgae have special properties such as antioxidant, antibacterial, antiviral and antifungal because they are rich in a variety of bioactive compounds. These bioactive compounds include some primary and secondary metabolites such as phytochromes (lutein and carotenoids), DHA, phenolic compounds, tannins, peptides, lipids, enzymes and vitamins, terpenoids and so on. Dietary fibre supplementation from macroalgae aids the maintenance and growth of beneficial intestinal flora and potential in reducing the risk of colorectal cancer. It was reported that regularly consuming some macroalgae can reduce the incidence of breast cancer [9]. Extracting industrial algal gums or compounds with antiviral, antibacterial or antitumour activities can be utilised as nutritional supplements and are applicable to humans or animals [2].

**Figure 1:**
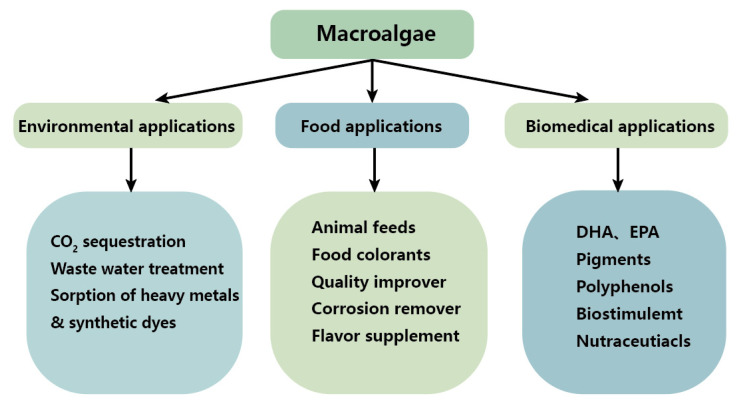
Comprehensive application of macroalgae.

This review mainly provides an overview of the distribution characteristics of macroalgae and their bioactive substances application in food, pharmaceuticals and environmental protection (Figure 1) [10,11]. However, wastewater treatment with macroalgae and their utilisation in food and medicine is unpredictable and anticipated. Additionally, this review further prospected the wastewater and waste gas treatment and utilization for macroalgae, which aims to provide a useful and informative basis for macroalgae production and resource utilization.

## 2. The Distribution Characteristics of Macroalgae

The seasonal change in seawater temperature is an important environmental factor for the change in macroalgae distribution. There is an evident seasonal alternation of macroalgae and there are differences in the number of species and biomass of macroalgae in varying seasons [12]. In general, springtime is when macroalgae vigorously grow and there are also various species of algae, and they can accumulate the most nutrients during this time.

**Figure 2:**
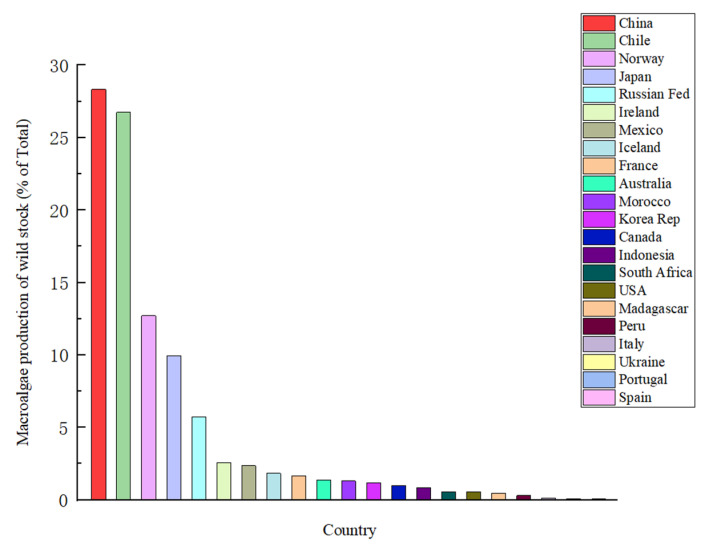
Distribution of macroalgae production in major countries around the world.

In general, the distribution areas of macroalgae includes horizontal, vertical and spatial and temporal regional differences. Horizontal distribution refers to the distribution along latitude, that is, zonal distribution; meanwhile, vertical distribution is the distribution along the tidal zone. Different environmental factors in different seasons influence the change in benthic macroalgal habitat, thus making the benthic macroalgal community change in species composition and structure [13]. Macroalgal communities have a key role in maintaining the stability of coastal ecosystems. The different geographical distributions of macroalgae create unique environments that provide necessary sites for epiphytic and symbiotic macroalgae and habitats for small marine animals (Figure 2).

### 2.1. Distribution of Macroalgae in Intertidal Areas

The intertidal zone refers to the intersection of terrestrial and marine ecosystems and is one of the most sensitive ecosystems in the biosphere [14]. Intertidal organisms are affected by tides and waves and show zonal distributions from high to low tide zones. Macroalgae are among the major communities that live in rocky reefs or gravel intertidal ecosystems. Macroalgae and their community structure is closely related to intertidal ecological factors such as substrate type, temperature, light, salinity, etc., and also directly related to human activities [15].

A clear vertical distribution of macroalgal populations in the intertidal zone with green algae gradually decreases from high tide to low tide, brown algae showing a zonal distribution in the middle tide zone, and red algae gradually increasing from high tide to low tide. The number of macroalgae species tend to increase from the high intertidal zone to the low intertidal zone. The high intertidal zone is subject to rapid changes in diurnal temperature and light and is mostly distributed with species of the green algae phylum. These species can tolerate strong light exposure and wet and dry changes in high and low tides twice daily. Brown algae are mainly dominant in the middle tide zone, supplemented by green algae with more species in spring, whilst most of these algae disappear in autumn. Meanwhile, red algae are mainly dominant in the lower part of the mid-tide and the low-tide zone. *Coralline algae*, *Sargassum* and *Grateloupia filicina* are the most common macroalgae, especially in areas near the low tide line where waves are crashing [16,17,18].

### 2.2. Distribution of Macroalgae in Mangrove Areas

Mangroves are a group of trees that grow in tropical and subtropical intertidal zones. They help purify seawater and maintain biodiversity. Mangrove areas are above the mid-tidal zone of the intertidal zone, and most of them are vast flat mudflats. Therefore, the algae growing in mangrove areas do not have evident characteristics of the vertical distribution of high, middle and low tidal zones.

Macroalgae distributions in mangrove areas are affected by changes in subsurface height, the distribution of higher plants and other growth environments. The clear vertical distribution of macroalgae on the mangrove plant body is an important difference between the distribution of them in mangrove areas and open intertidal areas. There is a clear difference in the ecological distribution of macroalgae in mangrove areas from that in open intertidal areas. Considering the shading effects of mangrove plants, the algae distributed under them are less exposed to light conditions and they are distributed less in quantity. According to the distribution characteristics, red algae prefer shaded and humid environments whilst green algae are suitable for better light conditions.

## 3. Nutritional Value of Macroalgae

Many macroalgae have high nutritional value, such as rich in vitamins, minerals and dietary fibre, and they can be taken as a low-calorie food. However, the extraction methods and/or processes of these bioactive substances from different species of macroalgae are different (Table 1). Some functions of macroalgae in food applications are popular in various Southeast Asian countries. Currently, the nutritional value of the polysaccharides and proteins of macroalgae is widely used in the food industry. It is applied as gels and thickeners and nutritional additives that can be used as animal feed.

Protein is an essential part of a healthy diet and the material basis of life. Protein is the basic organic substance that constitutes cells and is the essential nutrient for humans. Common food sources of high protein include animal protein, vegetable protein and soy products. Given the limited availability and high cost of animal protein sources, researchers are gradually focusing on plant protein as an alternative. They investigated polymerised essential amino acids, energy and minerals to form certain nutritional factors, thereby utilising plant proteins to improve the nutritional quality of products or as functional ingredients in plant chelated proteins (e.g., the use of soy and whey chelated proteins) [19,20].

Algae are rich in vitamins, minerals and proteins and have a high yield at a low cost. Considering that macroalgae have a high content of essential amino acids and unsaturated fatty acids, their proteins and lipids are more suitable to consume and have corresponding nutrition than other plant sources. Red algae have a large amount of protein, particularly the *Kappaphycus alvarezii* [21]. The protein concentrate (PC) of the macroalgae *K. alvarezii* was 62.3 ± 1.62%, thereby making the PC of *K. alvarezii* suitable for consumption as a cost-effective source of protein [21].

**Table 1:** Extraction and production of bioactive substances from macroalgae.

Macroalgae	Photosynthetic Pigments	Photosynthetic Rate	Algae	Process	Process Conditions	Generated Products	References
*Rhodophyta*	Phycoerythrin	20–1808.7 (μmol CO_2_/h) g/dry	*Gracilaria corticata*	SLE	Water, 12 h, 4 °C; UMFb, 200 kDa cut-off poly sulphone membrane	Pigments (R-PE and R-PC) Mineral-rich water	[22]
			*Gracilaria gracilis*	Fast pyrolysis	400, 500 and 600 °C, heating rate 50 °C, atmospheric pressure, inert atmosphere	Liquid	[23]
			*Kappa-phycus alvarezii*	Thermal acid hydrolysis	30–110 mM H_2_SO_4_, 30–120 min, 121 °C, pH 5.0 with NaOH	Agar	[24]
			*Gracilaria vermiculophylla*	Water Extraction	Pre-treatment (5% NaOH, 80 °C, 2 h); neutralization (1.5% H_2_SO_4_, 2 h); water extraction at boiling tempera-ture (90 min), 50 g/L	Agar	[25]
*Chlorophyta*	Chlorophyll a, b, carotene and Xanthophyll	30 to 1786 (μmol CO_2_/h) g/dry	*Ulva fasciata*	SLE	Water, 500 g/L, grinding	MRLE (mineral-rich liquid extract)	[26]
			*Ulva lactuca*	SLE	500 g/L, Water, crushing (biomass mechanically disintegrated into tiny particles) and filtration	Sap	[27]
			*Ulva lactuca*	Lipids extraction	Lyophilization, cell destruction with a high-pressure homogenizer, sequen-tial solvent extraction (3 times) with Folch solution	Lipids	[28]
			*Ulva lactuca*	SLE	0.25 M NaOH (optimized alkali con-centration), 50 g/L, 60 °C, 1 h	Proteins	[29]
*Phaeophyt*	Fucoxanthin	100–500 (μmol CO_2_/h) g/dry	*Laminaria digitata*	MAE	0.1 M HCl, microwave irradiation, 15 min, 90 °C; Alginate removal by 2% CaCl_2_; Fucoidan precipitation by ethanol	Fucoidan	[30]
			*Laminaria digitata*	Enzymatic hydrolysis	Enzymatic hydrolysis	Bioethanol	[31]
			*Sargassum muticum*	Ethanolic extraction and SC-CO_2_	Pilot plant extractor, 1 h, 50 °C, 10 and 35 MPa, 25 g CO_2_ min^−1^	Fucoxanthin	[32]
			*Saccharina japonica*	SLE	0.5–4.0 wt% sodium carbonate, 100 g/L, 40–80 °C, 60–120 min	Alginate	[33]

Note: SLE: solid–liquid extraction, MAE: microwave-assisted extraction.

### 3.1. Food Additives

#### 3.1.1. Flavor Supplements

Thermally processed flavourings have been widely used in various products such as soups, sauces, snacks and ready-to-eat foods. Meat flavour supplements have been studied and produced by processing animal proteins, mainly in the production of soybeans, wheat and other plant sources [34]. However, seafood flavour supplements produced from marine organisms such as fish, shrimp, and crab are having difficulty maintaining high production quality because of the high sensitivity of aquaculture organisms to lipid oxidation and the costly abnormal fat removal. Therefore, it is important to employ raw materials with high flavour and low cost to produce seafood flavour supplements. Moreover, macroalgae are abundant in various nutrients and have the potential to produce seafood flavour supplements because of their fresh flavour that has been used in soups and other kinds of foods.

Macroalgae contain taste-active amino acids such as aspartic acid, glutamic acid, arginine and lysine, which can be used to produce seafood flavour supplements [35]. Their protein can form the corresponding protein hydrolysate (PH) under the hydrolysis of bromelain. PH is the precursor of the heat-processed seafood flavour. Its special flavour substance after heat-processing can be used in the production of seafood flavour supplements. Laohakunjit [35] investigated the preparation of enzymatic bromelain seaweed protein hydrolysate (eb-SWPH) from *Gracilaria* sp. by using the response surface and the optimal hydrolysis conditions of pineapple protease for 3 h were 10%. With this method, the yield and degree of hydrolysis were 38.15% and 62.91%, respectively. In addition, the optimal hydrolysate contains three free amino acids, arginine, lysine and leucine. It is confirmed that the seafood essence has the flavour of grilled seafood after thermal processing by eb-SWPH.

#### 3.1.2. Quality Improver

The metabolites of macroalgae have been widely used in the food industry. Alginate is a high molecular weight structured polysaccharide obtained from macroalgae which forms viscous solutions when dissolved in water, therefore it is commonly used as a food quality enhancer during food processing. Algins mainly include agar and carrageenan from the red algae family and sodium alginate from brown algae, all of which have different commercial significance.

The agar is mainly extracted from the *Gelidium amansii* and *Gracilaria* sp. The agar consists of strong gel agarose and non-gel agarose lectins. Agarose consists of agarose units, that is the D-galactose moiety is bound to the β-1,4-glycosidic bond of 3,6-anhydro-L-galactose [36]. The greatest advantage of agar gels is their thermal reversibility. Depending on the species, agar gel melts at 85 °C or higher; however, it becomes a colourless and odourless gel after cooling [25]. Agar is mainly used as a thickening agent, emulsifier and gelling agent in the food industry, such as fruit jellies and canned meat. Given that agar does not melt in the oven, it is also used for filling and glazing pastries before baking [37].

Macroalgae carrageenan is an ionic polysaccharide that consists of galactose and sulfate distributed in polymer chains [38]. Carrageenan is the most commonly used algae gum in food processing and is a stabiliser and emulsifier in dessert mousse, salad dressings, ice cream and other various types of foods [39]. Adding carrageenan would affect the colour and texture of the bread during baking thereby giving it a special flavour. Carrageenan is also used in dairy products because of its unique ability to bind milk proteins. In addition, carrageenan keeps milk solids in suspension even at very low concentrations, thus stabilizing the dairy product [40]. Moreover, this prevents the separation of whey in cheese products and contributes to the formation of crystals in milk ice cream, thereby giving a smoother texture. Therefore, carrageenan is usually used in the production of cheese, cocoa and chocolate dairy products. Another area of application for carrageenan is the meat industry, where it is used in the production of ham, burgers, seafood and poultry products because of its water retention properties [41].

#### 3.1.3. Preservatives and Nutritional Supplements

Sodium alginate is often extracted from *Saccharina japonica* and *Thallus laminariae.* Similar to other algins, sodium alginate forms viscous solutions and gels when dissolved in water [42]. Sodium alginate can quickly absorb water and can chelate metal ions, and it is a part of the cell wall and intercellular matrix of all brown algae. Sodium alginate provides the elasticity and mechanical strength in macroalgae needed to survive in the ocean. Sodium alginate is widely used in the production of gels or as a viscosity modifier in the food industry to improve the appearance of bakery products and ensure the smooth texture of frozen foods. It is even dehydrated to enhance the appearance of dairy products and canned foods and water retention [43]. In addition, sodium alginate is often used as a stabiliser for beer foam [41].

Synthetic preservatives are the most common preservatives used in the food industry, but they may have harmful effects on the human body, thereby causing certain neurological disorders or immune abnormalities (e.g., ADHD, allergies, etc.). In addition, given the increasing focus on the naturalness of food products, the search for certain preservatives of natural biological origin has become a priority in the food industry. Macroalgae extract is a safe and environmentally friendly natural antioxidant that exhibits good antibacterial and anti-biofilm properties. Macroalgae extract has no side effects because it is rich in phenols, alkaloids and terpenoids such as butylated hydroxytoluene (BHT) and butylated hydroxyanisole (BHA) [44]. Studies have confirmed that adding antioxidants obtained from macroalgae can inhibit or delay lipid oxidation thus extending the shelf life of foods. Fucoidan and fucoidan polysaccharides extracted from brown algae such as *Sargassum natto* have been repeatedly used to develop biodegradable films [45,46,47].

**Figure 3:**
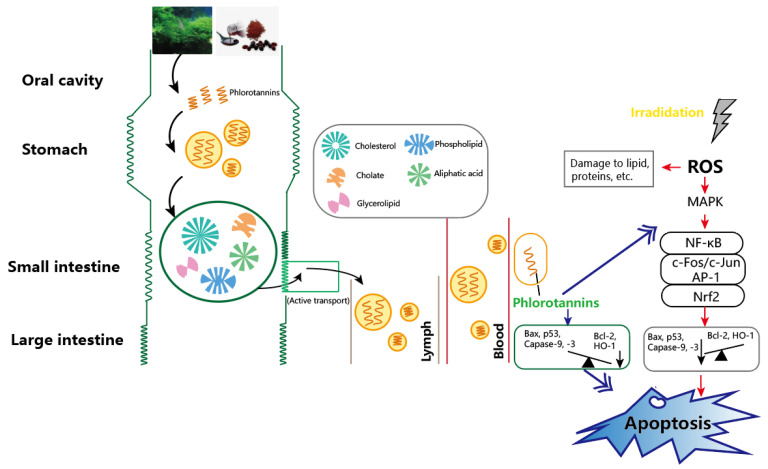
The mechanism of anti-radiation and antioxidant effects in humans from the active substances of macroalgae.

Macroalgae have a high nutritional value and are used as dietary supplements because of their active compounds that have antioxidant and anti-radiation properties. Reactive oxygen species (ROS) play an important role in various biochemical processes, such as vasodilation, neurotransmission, oxidative signaling and other activities. When the body is stimulated by large amounts of external radiation, the mitogen-activated protein kinase (MAPK) pathway is activated and induces the production of specific proteins, including nuclear factor-kappa B (NF-kB), c-Fos/c-Jun AP-1 complex, lipid raft protein caveolin-1 and other pro-inflammatory factors, which lead to cell damage. It has been shown that the active substances from macroalgae can act as potential blockers in the ROS metabolic pathway to reduce cellular damage and thus have the antioxidant effect after digestion or absorption (Figure 3) [48]. The inclusion of some macroalgae is beneficial to the human body and can also bring greater commercial value whether in people’s daily diet or in the food processing industry. 

### 3.2. Animal Feeds

Animal feeds are mainly produced from a mixture of various insects, fish and shrimp, eggs, meat and fishmeal in different proportions. Macroalgae as the sources of bioactive compounds in feed are considered as promising alternatives to conventional feed resources [47]. Macroalgae is a rich source of carbohydrates, protein, minerals, vitamins and dietary fibre in animal feed. Macroalgae has a relatively balanced amino acid structure and unique biologically active compounds, which can provide for the various nutritional needs of animals (Table 2) [49].

Adding macroalgae into poultry diets aims to increase the efficacy of feed absorption and can improve the quality of meat and eggs, whilst maintaining or improving poultry health. Green alga is often used as feed for broilers in food processing. Adding 3.0% of *Ulva lactuca* to broiler diets from the 12 to 33 days after egg hatching could improve breast muscle production and slaughter rate [50]. Brown algae by-products are often used as dietary supplements for broilers. Adding 0.5% of brown algae by-products to broiler diets can increase body weight, improve serum levels, immune responses and reduce mortality [51]. In addition, adding red macroalgae such as *Chondrus crispus* (1%) and *Gaudichadii* (2%) to poultry diets can also improve the egg quality. Meanwhile, adding *kappa algae* (1.5%) to broiler diets significantly reduced egg-laying age in laying hens and improved egg quality traits (e.g., egg production, egg weight and shell thickness) [52].

Moreover, macroalgae not only play a nutritional role in aquafeeds, but they also contribute to the overall health of the fish. It was reported that supplementing of *Ascophyllum nodosum*, *Porphyra yezoensis* or *Ulva pertusa* (5%) to red snapper (Pagrus major) diet increased body weight, feed utilisation and muscle protein deposition when compared to the normal diet [53]. Meanwhile, adding macroalgae (*U. lithospermum*, *P. yezoensis* and *U. pertusa*) to the diet can improve the immune and antioxidant response of European sea bass (*Dicentrarchus labrax*) without affecting growth performance [54].

**Table 2:** Application of macroalgae in animal feeds and its effect on product quality.

Livestock	Seaweed Species	Extract	Add the Amount	Main Biological Effects in the Animals	References
Barramundi (Lates calcarifer)	*Gracilaria pualvinata*	Dry	3%, 6%, and 9%	Increased levels of seaweed supplementation were negatively correlated with the serum triglycerides and cholesterol in the animals.	[55]
Buck and doe rabbits	*Ulva lactuca*	Fresh or dry	1% and 2%	Seaweed supplementation improved the reproductive performance of rabbits by improving the semen fertility characteristics of bucks.	[56]
Female pigs	Seaweed extract (No species mentioned)	Fresh or dry	180 mg laminarin and 340 mg fucoidan per kilogram feed	Seaweed extracts reduced the gene expression of pro-inflammatory cytokines in the colon of supplemented pigs after an experimental infection with Salmonella Typhimurium compared to animals receiving a basal diet.	[57]
Lohmann Lite hens	*Chondrus* *crispus* and *Sarcodiotheca* *gaudichaudii*	Fresh or dry	2% and 4%	The incorporation of seaweed in the diet of hens reduced the negative effects on body weight and egg production of a challenge with Salmonella enteritidis compared to birds receiving a basal diet.	[58]
Nile tilapia (Oreochiromisniloticx)	*Ulva* sp. (mixture of Ulva rigida 5% and 10% and ulva lactaca)	Fresh or dry	5% and 10%	Seaweed meal increased total carotenoid contents in the skin and enhanced immune responses in supplemented fish. Seaweed supplementation showed no effects on the growth performance or the organoleptic properties of the flesh with respect to fish receiving a basal diet.	[59]
Red tilapia (*Oreochronis* *miloticus*)	Fermented Enteromorpha prolifera	Dry	1%, 2%, 3%, 4% and 5%	Fermented seaweed had positive effects on the growth performance, the activity of the digestive enzymes and the immunity of the fish from the supplemented group with respect to control. The recommended optimum level of inclusion of this algal product in the diet of fish ranged between 3.7% and 4.19%.	[60]

## 4. The potential Medicinal Applications of Macroalgae

Different from terrestrial plants, macroalgae are abundant in active components such as polyphenols, polysaccharides, and amino acids, which are beneficial for the treatment of tumours, inflammation and cardiovascular diseases (Table 3). Therefore, the application of algae extracts in medicine is increasingly extensive and promising.

### 4.1. Polyphenols

Phenols are the main secondary metabolites of plants and are rich in various biological activities such as antioxidant, antitumour and immunomodulatory. Phloroglucinol is the polyphenol in most macroalgae, it has higher antioxidant and anticancer potential than the phenolic compounds of land plants (gallic acid and ellagic acid) [69].

It was reported that polyphenols from macroalgae (*Ecklonia cava)* protect cells from radiation-induced damage and oxidative stress. Three types of antioxidant-acting polyphenolic compounds, including mulberry pigment, catechol and epicatechin, isolated from *Ulva lactuca Linn* have strong scavenging activity of ABTs radicals, hydroxyl radicals and DPPH radicals [70]. Adding a large quantity of bromophenolic compounds from *Polysiphonia urceolata*, which has excellent antioxidant activity and inhibits α-glucosidase activity, can effectively reduce blood glucose in mice suffering from diabetes [71,72]. The free radical scavenging activity of polyphenolic fractions with molecular mass greater than 30 kD extracted from *Hizikia fusifarme* was higher than that of gallic acid, Vc and VE [73]. In addition, in vitro experiments with *H. fusifarme* polyphenols showed that the polyphenolic fractions were resistant to four types of tumour cells including HepG2, RAW264.7, HT29 and A-549 [74,75,76]. TDB isolated from the methanolic extract of *S. latiuscula (Harv) Yamada* also showed some antiviral effects against herpes simplex virus type 1 (HSV-1) [77]. 

### 4.2. Polysaccharides

In general, some polysaccharides synthesised by macroalgae can be used as algal cell components and energy storage substances for self-protection when subjected to external stimuli. Macroalgae polysaccharide residues mostly contain sulfate groups. The residues contain antioxidant and antiviral activities and are mostly related to the molecular size, structure, and the connection.

Polysaccharides extracted from green algae have anti-peroxidation and anti-hyperlipidaemia effects. It can effectively inhibit tissue abnormalities induced by D-galactosamine in mice [78]. The polysaccharide of Codium fragile can effectively inhibit the growth of human lung adenocarcinoma A549 cells [79]. *Chaetomorpha aerea* water-soluble galactan sulfate extract that consists of 18% arabinose, 24% glucose and 58% galactose show antibacterial activity against three Gram-positive bacteria including *Staphylococcus aureus* and its minimum inhibitory concentration is 40 mg/mL [80]. The polysaccharide of *Gloipeltis furcata* extracted from red algae has anti-tumour and mouse liver protection effects on tumour-bearing mice. Hence, when the physiological activity was improved, the tumour inhibition rate in the administration group reached 35.64% [81]. Dedhia isolated and purified three water-soluble sulfated polysaccharide fractions from *G. livida* (GLP-1, GLP-2 and GLP-3), and all with anticoagulant and antioxidant activities [82]. The purified *G. turuturu* polysaccharide fraction showed a minimum IC50 of 3.91 μg/mL against HSV-1 [83]. It was confirmed that polysaccharides from *Undaria pinnatifida* significantly inhibited the growth of human hepatocellular carcinoma cells HepG-2 with a tumour inhibition rate of 57.20% [84]. Wen studied the anti-inflammatory activity of polysaccharides from *S. horneri* and the results showed that polysaccharides from *S. horneri* can activate macrophages and produce strong anti-inflammatory effects [85].

### 4.3. Peptides and Other Substances

Functional peptides synthesised by macroalgae are stored in cells or extracellularly secreted and they exhibit biological activities such as antiviral, antibacterial and antioxidant. The peptide isolated and purified from *Ulva borealis* has anti-tobacco mosaic virus (TMV) activity. It was showed that the thermal stability is high when the molecular weight of the peptide is 23 kDa [86]. *Ishige okamurae* extracts could achieve anticancer effects by inhibiting the MMPs in HT1080 fibrosarcoma cells and its methanolic isolate fraction may be a potential inhibitor for MMPs [87].

In addition, the fatty acids, pigments and other active substances contained in macroalgae have higher commercial and beneficial value in the field of biomedicine. Macroalgae can synthesise and accumulate various long-chain polyunsaturated fatty acids, and the composition of *C. algae* has hypolipidemic activity. Their contents vary seasonally with a relative increase during summer and winter [88]. The free fatty acid composition of *Gloiopeltis furcata* also showed moderate antibacterial activity against *Mutans streptococci*, with MIC values ranging from 25 μg/mL to 50 μg/mL. Moreover, the monomeric compounds such as palmitic acid, cholesterol and phytol that was isolated and purified from *H. fusifarme* have antioxidant activities, with IC50 values ranging from 11.8 μg/mL to 135.2 μg/mL [89].

Macroalgae use their pigments to absorb light in the spectrum, such as fucoxanthin, astaxanthin, phycobiliprotein, etc. Fucoxanthin and Astaxanthin are two kinds of carotenoids with strong free radical scavenging activities and anti-inflammatory properties, and have significant effects to anti-cancer, anti-tumour and fat reduction [90]. Astaxanthin reduces plasma triglyceride and total cholesterol levels thereby limiting the increase in body weight and adipose tissue weight [91]. Aoi et al. found that adding astaxanthin (0.02% w·w^−^^1^ per 100 g) to an exercise mice model would increase their immunity by 14.5% [92]. Phycobiliproteins are a class of pigment proteins composed of phycobilin pigments, most phycobiliproteins for commercial production are from cyanobacteria (e.g., *Arthrospira*) and red algae (e.g., *Porphyridium*) [93]. The phycobiliprotein extracted from algae increases the activity of antioxidant enzymes in the human body to inhibit the production of ROS [94]. These data indicate that the active substances in macroalgae can be used as active ingredients in medicines or cosmetic/cosmeceutical formulations, such as in sunscreen or anti-aging creams.

## 5. Treatment of Wastewater and Waste Gas with Macroalgae and Their Potential Applications

With the development in the aquaculture, environmental concerns brought about by the discharge of aquaculture wastewater have become serious. Nitrogen, phosphorus, heavy metals and antibiotics contained in aquaculture wastewater can seriously pollute the aquatic environment. Macroalgae produce high nutritional value whilst fixing carbon dioxide, nitrogen, phosphorus and other nutrients through photosynthesis, thus, achieving ‘recycling’ and ‘resource utilisation’ of related nutrients in air and wastewater.

### 5.1. Wastewater Treatment with Macroalgae

Nitrogen and phosphorus are important elements that affect algal growth. Macroalgae use nitrogen in the form of inorganic nitrogen (NH_4_^+^-N) and some organic nitrogen (urea, amides and amino acids), and phosphorus in the form of PO_4_^3−^. In detail, algae mainly go through three pathways: oxidative phosphorylation, photosynthetic phosphorylation and substrate-level phosphorylation, and convert them into ATP and phospholipids. Algae use carbon dioxide, nitrogen and phosphorus as the main raw materials to carry out photosynthetic reactions in chloroplasts with the reaction formula:

106CO_2_ + 16NO_3_^−^ + HPO_4_^2−^ + 122H_2_O + 18H^+^ → C_106_H_263_O_110_N_16_P + 138O_2_

It was reported that using a moving bed bioreactor (MBBR-MA) to cultivate macroalgae (*Chetomorpha maxima*) for the removal of total nitrogen (TN) and total phosphorus (TP) from wastewater, and the average removal rates can reach 42.85 ± 5.5% and 83.7 ± 7.7%, respectively [95]. Studies have shown that some species of macroalgae can also remove dye from the wastewater, and cellulose isolated from macroalgae *Aegagropila linnaei* with MB adsorption capacity of 139 mg·g^−1^ [96].

The resource coupling of biomass and by-products after treating wastewater with macroalgae also has potential applications. It has been reported that the protein quality of wastewater-cultured green algae (such as *U. Lactuca*) is higher than that of the cow’s milk protein when mixed with an appropriate amount of skim milk powder, wheat and oat grains [97]. A colony containing two macroalgae *Rhizoclonium* sp. and *Ulotrix* sp. was isolated from the anaerobic digestion wastewater (ADPE). When the NH_4_^+^-N concentration of ADPE was 248 mg·L^−1^, the ammonium removal rate reached the maximum (30.6 ± 6.50 mg NH_4_^+^-N L^−1^d^−1^), and the contents of carbohydrate and protein were 42.8–54.8% and 43.4–45.0% [98], respectively. *Rhizoclonium* sp. and *Ulotrix* sp. communities cultivated with ADPE had higher protein content and were suitable as protein feeds when compared to other common feeds. Meanwhile, the chlorophyll content can reach 40–45% under high ammonium conditions of 248 mg·L^−1^. Moreover, pigments are suitable for food additives and the chlorophyll content produced was positively correlated with the nitrogen concentration in the medium [99]. Using *Spirogyra* sp. for treating primary wastewater (PW), secondary wastewater (SW) and concentrated wastewater (CW), and the results showed that the biomass content from CW culture is relatively higher than that of the PW and SW media. The corresponding protein, carbohydrate and lipid obtained after treatment accounted for 16.7%, 55.0% and 10.0%, respectively [99].

### 5.2. Carbon Sequestration with Macroalgae and Their Resource Utilization

**Figure 4:**
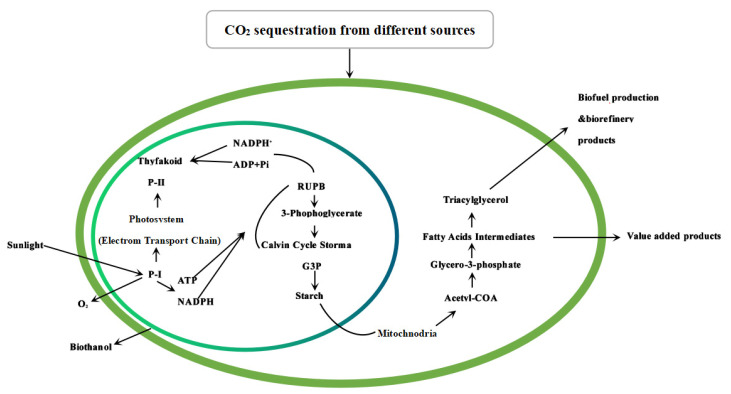
A schematic mechanism of macroalgae to fix CO_2_ and produce bioenergy.

Macroalgae can capture inorganic carbon and CO_2_ through photosynthesis and incorporate them into various macromolecular metabolites and other biochemical components including RNA (ribonucleic acid) and DNA (deoxyribonucleic acid) [100]. There are three main ways for algae to absorb inorganic carbon: (1) use extracellular Carbonic Anhydrase (CA) to convert bicarbonate to carbon dioxide; (2) directly absorb carbon dioxide through the plasma membrane; (3) directly absorb bicarbonate through active transporters in the membrane. Figure 4 shows a schematic mechanism of algae to fix CO_2_. The photochemical reaction is composed of two photosystems, namely photosystem I and photosystem II. Through a transport system, electrons released by the light-driven oxidation of water molecules are transferred to NADP^+^ and it is reduced to NADPH. Protons in the matrix are pumped into the thylakoid lumen as the consequence of electron transport, and then creating a transmembrane proton gradient thereby driving phosphorylation of ADP to generate ATP. In the dark reaction stage, ATP and NADPH fix carbon dioxide into glucose through a light-independent reaction, which is then converted into various high-value foods and drug metabolites.

As we know, carbon fixation capacity of algae is limited by their species, light, temperature, etc., because the above factors affect carbon fixation by influencing the photosynthetic efficiency. Therefore, the suitable algal species should be selected, and proper environmental conditions should be created for their growth and accumulation of metabolites during wastewater treatment. Light intensity is an important factor affecting photosynthetic carbon sequestration in macroalgae. Increasing light intensity below the light saturation point promotes photosynthesis, whereas light inhibition is followed by a decrease in carbon sequestration capacity. Different species of macroalgae have different temperature tolerances, and most of them have an optimum growth temperature of 15 °C–30 °C [101]. Inhibition of CA activity in algae reduces the conversion of HCO_3_^−^ to CO_2_, thus, leading to a decrease in CO_2_ required for carboxylation reactions thereby ultimately weakening photosynthesis [101]. In addition, UVR (Ultra-Violet Radiation) affects protein synthesis in PSII, inhibits rubisco enzyme activity, causes DNA damage and reduces pigment content, thus affecting the carbon sequestration capacity of macroalgae [102].

In a word, macroalgae are also currently considered the most promising third-generation biomass energy source, which are further converted into metabolic products of polysaccharides, polyphenols and proteins (amino acids, etc.) based on the purification of the marine ecosystem and CO_2_ fixation with important values through bio-refinement.

## 6. Conclusions

Macroalgae are economically valuable biomass resources that are widely applied in food, pharmaceuticals and environmental remediation. Different geographical distributions of macroalgae create their unique bioactive applications in different industries. The functional food ingredients and other molecules that are naturally found in macroalgae further provide better choices for food and animal feed. In addition, macroalgae are rich in active components which are beneficial in treating tumours, inflammation and cardiovascular diseases, and they have important potential in wastewater and waste gas (CO_2_) treatment. Therefore, it is desirable for us to value nutrient production from macroalgae whilst fixing carbon dioxide, nitrogen, phosphorus and other nutrients in wastewater through photosynthesis to achieve resource utilisation of related nutrients from CO_2_ and wastewater in the future.

## Figures and Tables

**Table 3:** Polysaccharides, polyphenols, peptides from macroalgae and their active applications.

Macroalgae	Polysaccharides	Polyphenols	Peptides	Biological Activity	Applications	References
*Chlorophyta*	Ulvan	Alkaloids	NbrbcS1-1, NbrbcS1-2	Anti-tumour, anti-oxidant anti-thrombolytic immunal modulation, anti-influenza, and anti-coagulant	Tissue engineering	[61,62,63]
*Rhodophyta*	Carrageenan	Terpenoids	FQIN [M(O)] CILR, TGAPCR	Anti-coagulant, plateletaggregation inhibition, anti-viral, anti-tumuor activity	Anti-oxidant, drug delivery	[61,63,64]
	Porphyran	Phenolic compounds		Degraded and untreated porphyran possesses scavenging-free radical activity and functions as a reducing power	Cytotoxic, drug delivery	[61,63,65,66]
	Agar	Rutin		Extracted for gelling and stabilising capabilities, anti-inflammatory	Anti-oxidant, drug delivery	[62,63]
*Phaeophyceae*	Alginate	Catechol	Aβ peptides	Commercial alginate salts have immunal modulation properties	Drug delivery, anti-fungal, anti-tumour	[61,62,63]
	Laminarin	Hesperidin		Anti-tumour, anti-inflammatory, immune-stimulatory, anti-coagulant and anti-oxidant activity		[63,67]
	Fucoidan	Fucoxanthins		Anti-viral, anti-tumor, immune-stimulatory, anti-oxidant, anti-inflammatory, anti-coagulantand, anti-thrombotic activity. Ability to reduce cholesterol, triglyceride and LDL-C, andincrease HDL-C. Gastric protection (e.g., antiulcer, anti-adhesion for Helicobacter pyroli), protection against urinary tract, kidney and liver diseases.		[63,67,68]

## Data Availability

Data is contained within the article.
